# First Person Experience of Body Transfer in Virtual Reality

**DOI:** 10.1371/journal.pone.0010564

**Published:** 2010-05-12

**Authors:** Mel Slater, Bernhard Spanlang, Maria V. Sanchez-Vives, Olaf Blanke

**Affiliations:** 1 Institució Catalana Recerca i Estudis Avançats (ICREA), Universitat de Barcelona, Barcelona, Spain; 2 Facultat de Psicologia, Universitat de Barcelona, Barcelona, Spain; 3 Department of Computer Science, University College London, London, United Kingdom; 4 Departament de LSI, Universitat Politècnica de Catalunya, Barcelona, Spain; 5 Institut d'Investigacions Biomèdiques August Pi i Sunyer (IDIBAPS), Barcelona, Spain; 6 Brain-Mind Institute, Ecole Polytechnique Fédérale de Lausanne (EPFL), Lausanne, Switzerland; Macquarie University, Australia

## Abstract

**Background:**

Altering the normal association between touch and its visual correlate can result in the illusory perception of a fake limb as part of our own body. Thus, when touch is seen to be applied to a rubber hand while felt synchronously on the corresponding hidden real hand, an illusion of ownership of the rubber hand usually occurs. The illusion has also been demonstrated using visuomotor correlation between the movements of the hidden real hand and the seen fake hand. This type of paradigm has been used with respect to the whole body generating out-of-the-body and body substitution illusions. However, such studies have only ever manipulated a single factor and although they used a form of virtual reality have not exploited the power of immersive virtual reality (IVR) to produce radical transformations in body ownership.

**Principal Findings:**

Here we show that a first person perspective of a life-sized virtual human female body that appears to substitute the male subjects' own bodies was sufficient to generate a body transfer illusion. This was demonstrated subjectively by questionnaire and physiologically through heart-rate deceleration in response to a threat to the virtual body. This finding is in contrast to earlier experimental studies that assume visuotactile synchrony to be the critical contributory factor in ownership illusions. Our finding was possible because IVR allowed us to use a novel experimental design for this type of problem with three independent binary factors: (i) perspective position (first or third), (ii) synchronous or asynchronous mirror reflections and (iii) synchrony or asynchrony between felt and seen touch.

**Conclusions:**

The results support the notion that bottom-up perceptual mechanisms can temporarily override top down knowledge resulting in a radical illusion of transfer of body ownership. The research also illustrates immersive virtual reality as a powerful tool in the study of body representation and experience, since it supports experimental manipulations that would otherwise be infeasible, with the technology being mature enough to represent human bodies and their motion.

## Introduction

Normally when something strikes our body we feel it at the same place that we see it. When normal correlation between two sensory streams is changed, for example, by seeing a plausibly located rubber hand touched while simultaneously feeling the touch on our out-of-sight real hand, the brain apparently engages in a re-evaluation of probabilities and assigns ownership to the visible rubber limb [1], [2]. These methods have also been used to produce illusions of body morphing, adding supernumery limbs to the body [3], [4], [5], [6], and out-of-the-body experiences [7], [8], [9]. In conjunction with brain-imaging techniques these manipulations can provide insight into the brain areas involved in body representation, for example as in [10]. While the vast majority of work in this field has shown that it is possible to incorporate physical objects or video images of these into the body representation, it has also recently been shown that the same methods work with entirely virtual objects [11], [12], [13].

The examples of out-of-the-body experiences provide indirect evidence that these illusions might apply to the whole body rather than only to body parts. There is also evidence that ownership can be attributed to a manikin that appears visually to substitute the person's real body as seen through head-mounted displays coupled to a video camera oriented down at the manikin body [14]. These out-of-the-body and the manikin experiments employed synchronous visuotactile stimulation – the illusory visual body was seen to be tapped or stroked in the same place as the real body was felt to be stimulated. When there is asynchrony between felt and seen touches changes in ownership do not occur or are less prominent compared to the case of synchrony between both stimuli [13].

The experiment reported here is the first that shows that ownership can be transferred to an entirely virtual body, using an experimental design that separates perspective position from visuotactile stimulation. We found that when perspective position is included as a factor in the experimental design the importance of visual-tactile synchronization diminishes in comparison to what would be expected from the literature.

## Results

### Overall Design

**Figure 1:**
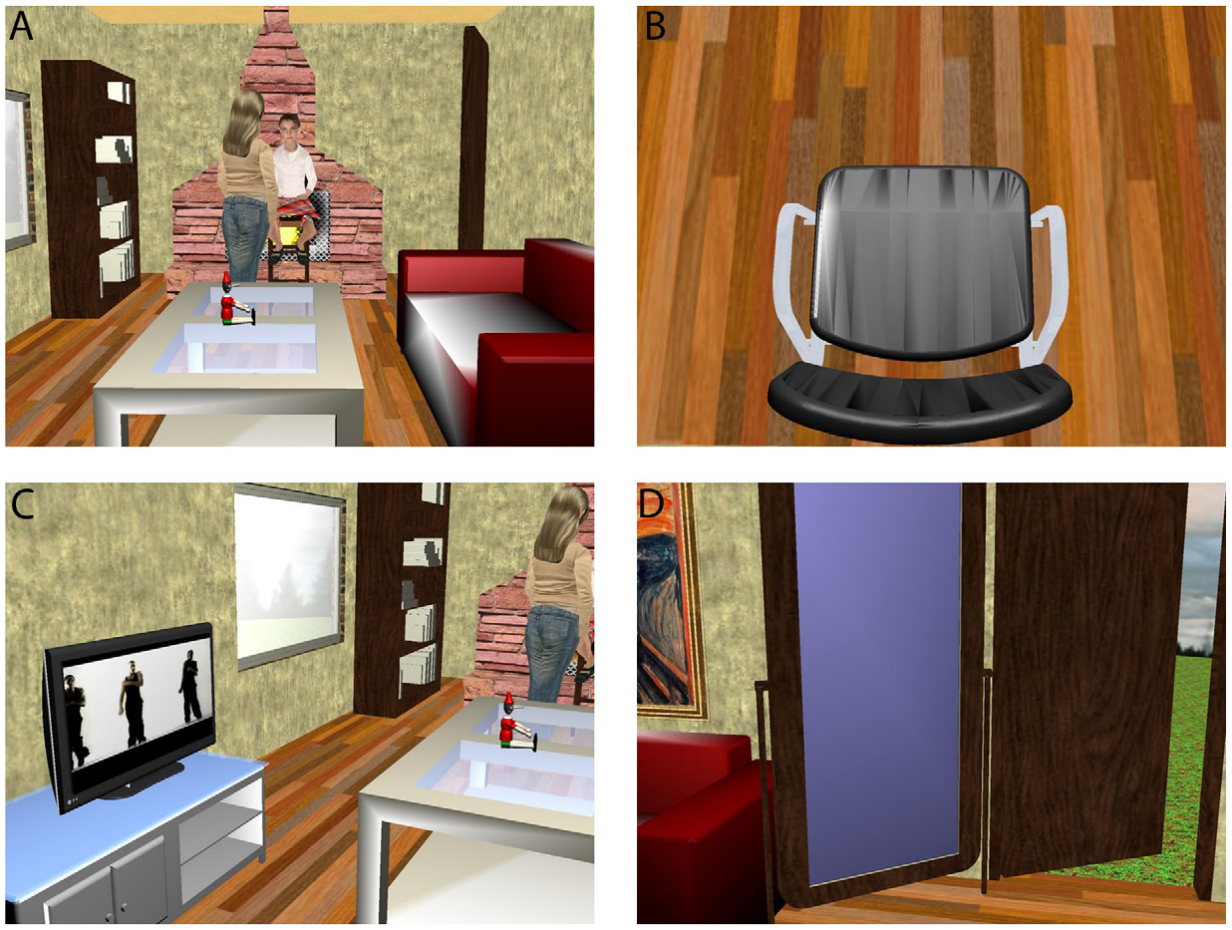
The scene. The scene that the participants entered was a room approximately the same size as the real room in which they were located. (A) There were two female characters at the other end of the room, a standing woman who could be seen stroking the shoulder of a seated girl, and a fireplace behind. (B) Looking down at himself a participant would see an empty chair. (C) To the participant's left was a TV showing a real-time music video. (D) To the right were a mirror frame and a door opening to a field.

**Figure 2:**
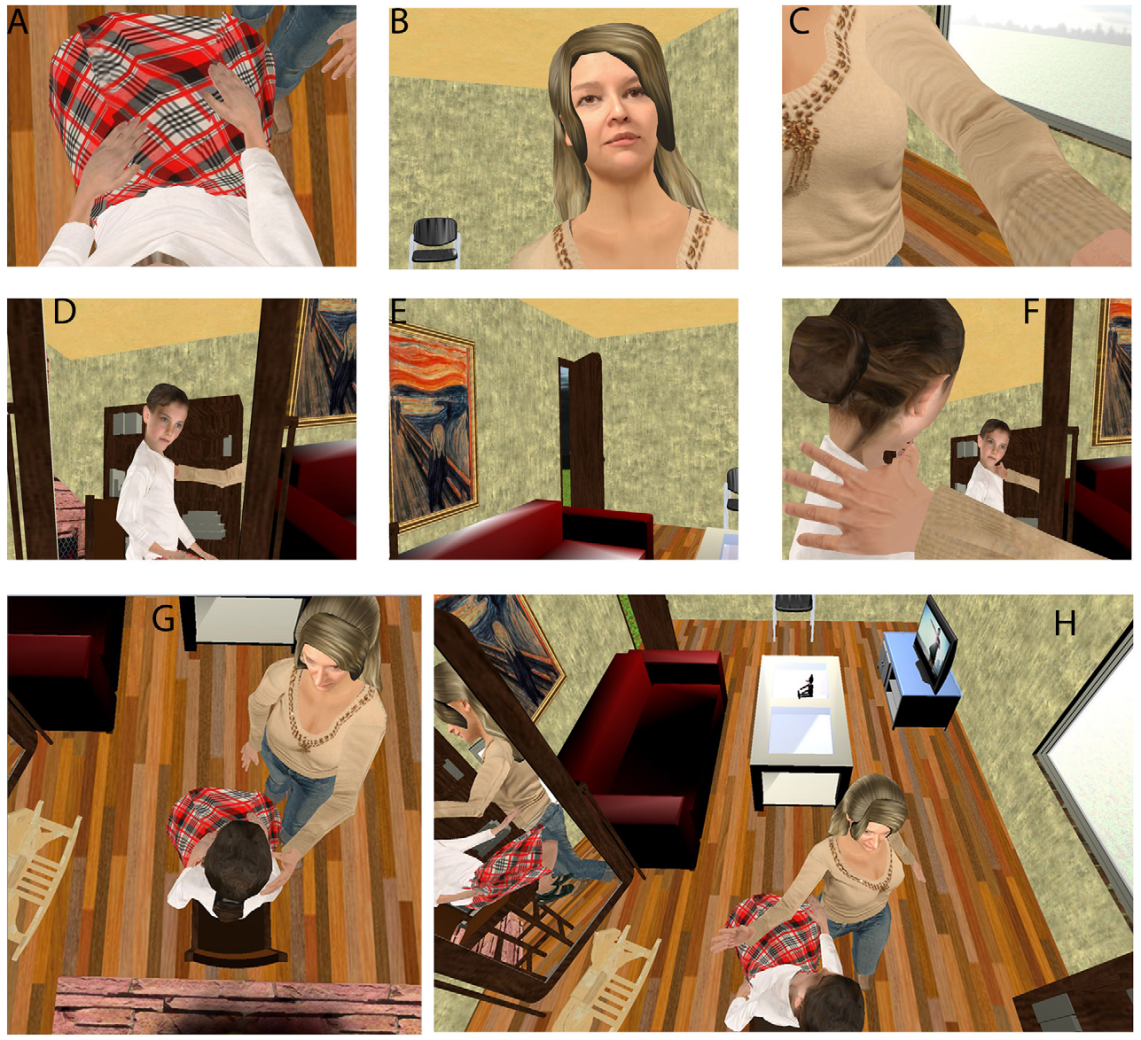
Participants were transferred to the other side of the room. (A) In the 1PP condition their body was substituted by that of the girl's (white shirt), and when looking down at themselves they would see her body. (B) Looking up they would now see that the woman (brown sweater) was standing by them. (C) The woman stroked their shoulder. (D) Looking left they would see the reflection of the girl and the woman in a mirror. (E) They were seeing the room and hearing the sounds from the TV from the perspective of the opposite side than in the first two minutes. (F) In the 3PP condition they would be located to the right of the girl, and so see her and her reflection in the mirror – in the case shown with her head moves synchronized with their own head moves. (G) Later the viewpoint shifted near to the ceiling and the woman continued to stroke the shoulder of the girl, but the participant did not feel this. (H) Suddenly the woman struck the girl three times around the face - the wide-field-of-view in this image corresponds more precisely to what the subject would have seen.

There were 24 male participants recruited for our study. They were seated, and entered into the virtual reality through a wide field-of-view head-tracked, head-mounted display and stereo headphones. The scene in which they were located is shown in Figure 1. They were asked to visually explore this scene for 2 minutes after which their viewpoint was transported to the other side of the room to where two female virtual characters were located, a seated young girl and a standing woman (Figure 2).

What they experienced then depended on which of the combinations of three binary factors they had been assigned by the experimental design (Table 1). *Perspective* was either first person (1PP, Figure 2A) or third (3PP, Figure 2F) with respect to the seated girl. *Movement* refers to whether the observed head movements of the virtual girl were synchronous with those of the subject (MS, Figure 2D, 2F) or asynchronous (MS′). *Touch* refers to whether the subject felt synchronously (TS) or asynchronously (TS′) touched on his shoulder when the standing woman stroked the shoulder of the seated girl (Figure 2C, 2D, 2F).

**Table 1:** Allocation of Participants to the Experimental Factors.

	Subject number:																							
Factor:	1	2	3	4	5	6	7	8	9	10	11	12	13	14	15	16	17	18	19	20	21	22	23	24
P	0	1	0	1	1	0	1	0	0	1	0	1	1	1	0	0	1	0	0	1	1	0	1	0
M	1	1	1	0	1	0	0	0	1	0	0	1	0	0	1	0	1	0	0	0	1	1	1	1
T	0	0	1	0	0	1	1	1	1	0	0	1	0	1	0	0	1	1	0	1	1	1	0	0

For example subject 1 was allocated to the condition P′, M, T′; subject 9 to the condition P′, M, T.

After almost 7 minutes of this period that included occasional shoulder stroking, the viewpoint of the participant was lifted upwards towards the ceiling, looking down on the scene below (Figure 2G) during which time the shoulder stroking continued but unaccompanied by physical sensations. Suddenly the standing woman was seen to hit the seated girl around the face (Figure 2H). After this the viewpoint translated downwards again, there were some more (felt) shoulder strokes, and then the experimental trial was terminated. The full sequence of events that occurred is shown in Table 2.

**Table 2:** Sequence of the Events During the Experiment.

Variable Name	Event Description	Time to next event (s)	Cummulative time (s)
	Baseline: Experiment Starts with participant seeing the girl and woman across the other side of the room	120	120
*across*	Move across the room to enter the girl's perspective (1PP) or to the right of the girl's perspective (3PP)	5	125
*stroke_1*	First stroke by the woman on the girl's shoulder	415	540
	Perspective Shifts to the ceiling – girl and woman seen below	5	545
	First arm stroke seen from ceiling position	45	590
*duringS*	The woman slaps the girl*	50	640
*down*	The perspective shifts back down to the girl	20	660
*stroke_n*	The final arm stroke	31	691
	The view moves back to the original perspective, and this continues for a final 30s.	30	-

*The event *beforeS* (before slap) was taken as 7s before the actual slap.

### Questionnaire Results

**Figure 3:**
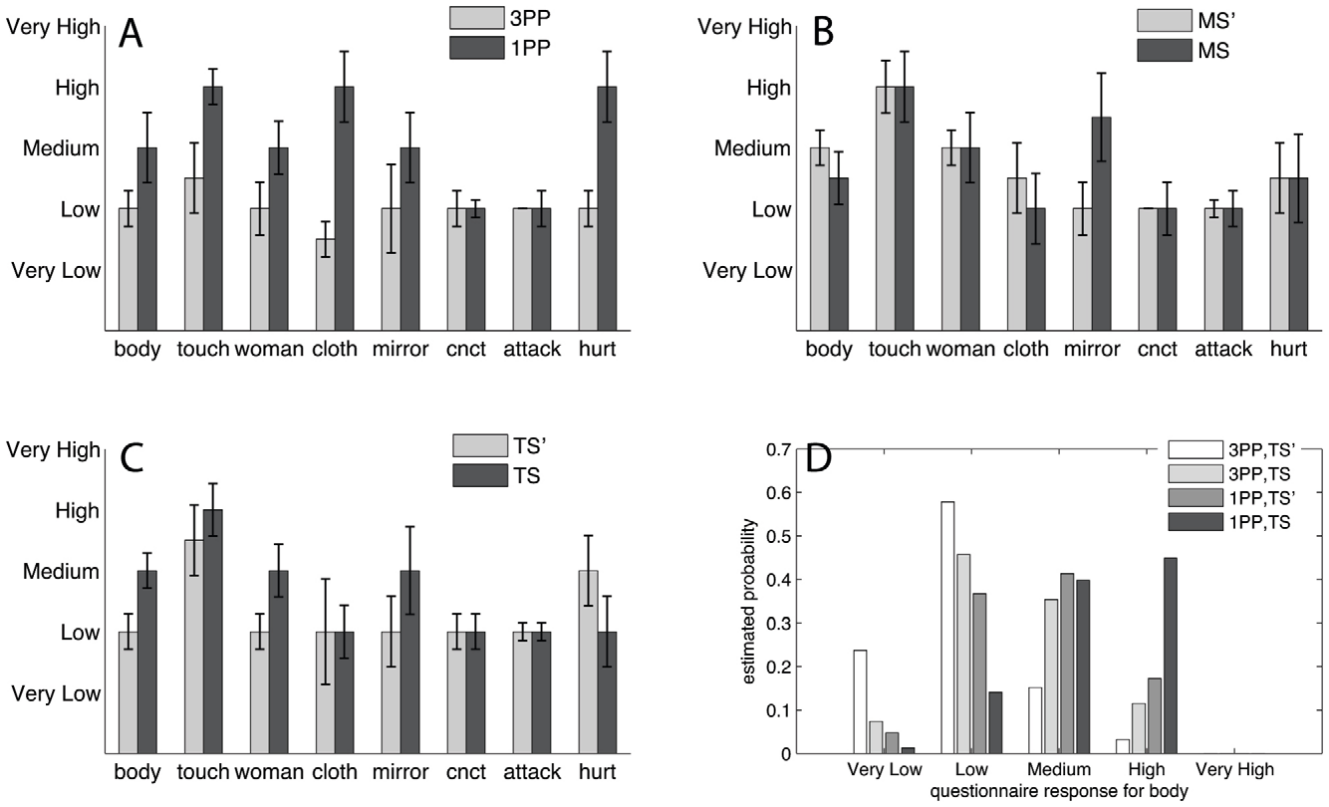
Questionnaire responses for the main effects. (A–C) show the means and standard errors of the questionnaire responses by each of P, M and T. Using proportional-odds cumulative logit models the notable significance levels are for P (*body*, p = 0.031; *touch*, 0.023; *woman*, 0.033; *cloth*, 0.003; *hurt*, 0.046), and T (*body*, p = 0.095; *touch*, 0.085; *woman*, 0.024). The model fits were good, with the highest deviance being 29.8 on 25 d.f. Panel (D) shows the estimated probabilities for the questionnaire responses for *body*, for four cases: for third person (P3, disembodiment) and for asynchronous (TS′) and with synchronous touch (TS), and for first person (P1, embodiment) again comparing TS′ with TS. In each case M = MS (the graph is almost identical for M = MS′). There were no scores of 10 in these responses which accounts for the low estimated probability of ‘Very High’.

Immediately after the experience in the virtual reality, a 13-item questionnaire was answered by the participants (Questionnaire S1). Eight of these questions related to the issue of body ownership (Table 3). Perspective gives the clearest set of responses (Figure 3A), where the mean (and median) score for 1PP is always greater than or equal that for 3PP on each of the questions. Movement appears to have no particular effect, and synchronous touch has an effect on some of the variables. From the fitted models estimates of the probabilities of the questionnaire scores for four combinations of the factors were obtained and are shown in Figure 3D. These data show that the most important factors leading to the temporary subjective illusion of ownership of the virtual body are the participant's perspective (i.e. in the girl's body, 1PP) and touch (TS), concordant with a recent account of self-consciousness [15]. Our data also show that apparent head-movement synchrony was least important for the body ownership illusion.

**Table 3:** Questions relating to body ownership and their labels used in the text and figures.

*body*	How much did you feel that the seated girl's body was your body?
*touch*	How strong was the feeling that the woman you saw was directly touching you on the shoulder?
*woman*	How strong was the feeling that the touch you felt was caused by the woman that you saw?
*cloth*	How strong was the feeling that you were wearing different clothing, from when you started the experiment, while you were in the part of the room where the standing woman was located?
*mirror*	How strong was the feeling that the body of the girl in the mirror was your body?
*cnct*	When you were looking down from above how much did you feel a strong connection with the seated girl as if you were looking down at yourself?
*attack*	When the standing woman hit the seated woman, how much did you feel this as if this was an attack on your body?
*hurt*	After you returned from looking down from above how much did you feel that the standing woman might hurt you?

### Heart Rate Deceleration

**Figure 4:**
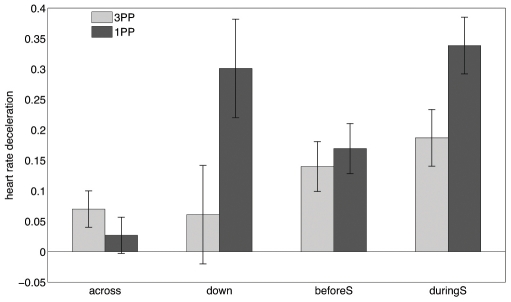
Means and standard errors of the Heart Rate Deceleration data. The figure shows the means and standard errors for HRD after four events: *across*: 0.5s after initially arriving at the other side of the room; *down*: 0.5s after descending from above; *beforeS*: 7s before the slap; *duringS*: 2s into the slap sequence. 1PP was significantly greater than 3PP on *down* (0.028) and *duringS* (0.034). The ANOVA fits satisfied the requirement of normally distributed residual errors using the Jarque-Bera test [37], except for 1PP on *down*, where a variable transformation was found to obtain normality.

We measured heart rate deceleration (HRD) in response to the woman slapping the girl, a parameter that has been associated with reports of aversive stress in the context of picture viewing [16]. We calculated the negative of the slope of heart rate change during the first 6s after the event in question. The greater this value the greater the initial deceleration and the greater the degree of aversive stress (p588). We consider HRD for two pairs of events (Figure 4). After the *down* transition (Table 2) the participants who perceived from the girl's perspective (1PP) showed a significantly greater HRD than the participants who perceived the scene from the displaced perspective (3PP). The same analysis was carried out for a control period (*across*) and revealed no significant difference between these groups of participants. Similarly, we found that during the slap (*duringS*) the 1PP participants had a significantly greater HRD than the 3PP participants, but for *beforeS* there was no significant difference. Amongst the three factors considered in this experiment only Perspective had a significant influence on the HRD response.

There is, furthermore, consistency between physiological responses (HRD) and the subjective questionnaire responses. During the slap (*duringS*) and after the period of being in the elevated position (*down*) the HRD was significantly positively correlated with a feeling of the participant's own body being attacked (*attack*), the feeling that they might be hurt by the woman (*hurt*) and body ownership (*body*). However, there were no significant correlations between any of the questionnaire responses and HRD for the control periods (*beforeS* and *across*). The full set of correlations and significance levels is in Table 4.

**Table 4:** Correlations Between Questionnaire Responses and Heart Rate Deceleration (significance levels in brackets).

Event	cnct	attack	hurt	body	touch	mirr	woman	clothing
*across*	−0.02	0.06	−0.35	−0.35	−0.33	−0.38	−0.35	−0.36
*beforeS*	0.03	0.21	0.15	0.11	0.06	−0.06	−0.09	0.10
*duringS*	**0.43**(0.038)	**0.43**(0.04)	**0.58**(0.00)	**0.39**(0.06)	0.25	0.04	0.10	**0.55**(0.00)
*down*	0.20	**0.40**(0.05)	**0.49**(0.02)	**0.61**(0.00)	**0.43**(0.04)	0.25	0.35	0.31

## Discussion

Our study extends earlier results that used simpler video and virtual reality technology [7], [8], [9], [14]. These suggested that synchronous touch (TS) and 1PP [17], [18] to be crucial factors for the sense of ownership. Importantly, these previous studies only manipulated a single one of the three factors (Perspective, Movement, Touch). Our results suggest that when all three factors are considered together that perspective, specifically first-person perspective, clearly dominates as an explanatory factor for subjective and physiological measures of ownership. The latter provides a particularly powerful result, since participants were responding to witnessing the girl being slapped while they were in an elevated position even without any synchronous touch. The 1PP participants, i.e. those who *earlier* had been in the first person perspective with respect to the girl's body, had a significantly greater physiological response than those who had earlier been in a spatially close but distinct virtual perspective (3PP). Moreover, stronger heart rate deceleration was positively correlated with the feeling of body ownership and the feeling of being attacked or hurt.

The minimal contribution of the specific type of agency that we investigated (MS′ compared to MS) seems to be in conflict with previous studies that suggested the importance of motor cues for the sense of self [19], [20]. We note that previous studies have focussed almost entirely on agency manipulations with respect to the upper extremity and have been carried out in isolation from perspective and touch manipulations (see [21] for an exception), making problematic any direct comparison with our results. However, considering our two questions that relate directly to body ownership, (*body* and *mirror* from Table 3) participants in condition MS were more likely to give a higher score to *mirror* than to *body* compared to those in MS′. A plot of the scores is shown in Figure S1. There are only 2 out of 12 cases for those in condition MS where *mirror* < *body*, and only 1 out of 12 cases for those in condition MS′ where *mirror* > *body*. The correlation between these two sets of scores for those in condition MS (r = 0.91, p<3.5×10^−5^) is greater than for those in MS′ (r = 0.71, p<0.01). Analysis of covariance of *mirror* on condition M with *body* as a covariate suggests that the regression line of *mirror* on *body* has greater slope in condition MS than in condition MS′ (p = 0.06). The same analysis has stronger support for there being two positively sloped parallel lines with the one for MS having a greater intercept than for MS′ (p = 0.0093). This suggests that the synchronised head movement did, after all, make some difference – resulting in those participants in condition MS giving higher scores to the question *mirror* than to the question *body*.

It could be argued that the amount of synchronous visuo-tactile stimulation was less than what is normally used to induce the rubber hand illusion. Yet according to [22] the RHI can be generated in about 80% of people with less than 15s of stimulation, provided that the rubber hand and real hand are close to one another (15–18cm). Moreover in our setup, unlike that of the RHI, the participant was not required to continually look at the actual point of contact between the virtual hand and shoulder. For most of the time they looked up towards the virtual woman, and would see her arm move up and down in synchrony with feeling of the strokes, or see the same in the mirror reflection. Finally, we note that continuous stroking may not be necessary to induce the illusion. For example [23] showed that the RHI could be ‘topped up’ by occasional sequences of stroking with periods of no stroking in between. See Methods for further details of the stroking sequences in our experiment.

Our experiment includes that of Lenggenhager et al. [7] as a special case. The essence of their setup was to manipulate ownership by a 3PP self-representation, that was touched asynchronously or synchronously, comparable to our 3PP and TS′ compared with 3PP and TS. Figure 3D shows that with respect to the questionnaire responses the estimated probability of the response being in the Very Low category was much greater in the asynchronous touch than in the synchronous touch condition. Figure S2 gives the equivalent graph for the questions *touch* and *woman*, which have greater probability estimates for the Medium to High questionnaire response in the synchronous compared to the asynchronous condition. There are no significant effects for T in the case of the HRD.

Our experiment also includes that of Petkova and Ehrsson [14] as a special case. Their main setup was to also manipulate ownership by a 1PP self-representation (a manikin) that was touched either asynchronously or synchronously – similar to our 1PP and TS′ (girl's perspective and asynchronous stroking) and 1PP and TS (girl's perspective but synchronous stroking), respectively. Figure 3D shows that the responses to the question *body* supports the idea that synchronous stroking enhances ownership of the 1PP perceived virtual body. The same is true for the other questionnaire responses (Figure S2). However, HRD is not affected by T.

In [14] there was a significantly different skin conductance response when the manikin body was threatened in the synchronous compared to the asynchronous touch condition. However, the HRD measure that we use does not measure the same type of response as skin conductance. The latter measures arousal, the valence of which is unknown. HRD in particular has been proposed to measure the degree of aversion to images [16]. In fact in our study there was no significant difference with respect to skin conductance on seeing the slap between the 1PP and 3PP conditions or TS and TS′ conditions - it is simply an arousing event to see someone slapped. However, there was significantly greater aversion (as quantified by HRD) for those in the 1PP condition, we speculate because they had a greater degree of association with the body that was seen to be slapped.

Additionally there is a critical difference between our experiment and that of Petkova and Ehrsson [14] where cameras on the manikin's head were in a fixed position, looking down at the manikin's body, and therefore the experimental subjects had to have their head fixed in the same orientation. In our setup the real-time head-tracking ensured that the act of looking down involved motor acts and corresponding perceptual changes comparable to physical reality. This may be why in Petkova's and Ehrsson's setup synchronized touch was a critical factor to achieve changes in ownership but it was less important in our experiment. This also ties in with a recent observation that the strong illusion of being in the place depicted by the virtual reality [24] occurs when sensorimotor contingencies for perception [25], [26] are similar to those of physical reality, that is, when a participant can use their body for perception in much the same way as normally [27]. Sensorimotor contingencies endow ‘place-ness’ to virtual space and the objects within it, and a unique and highly special object is one's own body. When the virtual body is perceived to be in the same place as where the real body should be, perhaps this provides very strong evidence for the brain to generate the illusion that the virtual body is one's own. This finding was unexpected in comparison with previous results that have emphasized the importance of visuotactile synchronization.

The experiment had the unusual goal of attempting to generate a body ownership illusion where the virtual body did not visually resemble the real body of the participants, and was not even the same gender. The reasoning was that if it were the case that the illusion could be generated in these circumstances then it should also be possible in a range of other less extreme situations - without gender change, with less of a radical difference between the participant's own body and the virtual body. Gender categorization is known to be persistent. For example, in [28] an experiment is reported that shows that racial categorizations can be eliminated in favor of categorization by membership of a cross-racial coalition. When the same technique was applied to gender categorization the effect of gender could not be extinguished. That experiment therefore provided an illustration that top-down cognitive manipulations could reduce the effect of race, but not of gender.

In contrast our experiment used mainly bottom-up sensory stimuli – visual, tactile, vestibular and proprioceptive signals and their correlations. This would lead us to think that with respect to the issue of body ownership, bottom-up perceptual signals play a more dominant role than top-down processing. Botvinick and Cohen [1] proposed that visuo-tactile integration was sufficient to generate the feeling that proprioception had shifted to the rubber arm, while Armel and Ramachandran [2] went further to postulate that such bottom-up sensory integration between vision and touch was sufficient to generate the illusion since it was shown to operate even with a neutral object such as a table. However, this result was not reproduced by Tsakiris and Haggard [29] who found that the illusion did not occur when a wooden stick was used in place of a rubber arm, nor did it occur when there was postural incongruence (the left hand stimulated with the rubber hand being a right hand). This led them to conclude that bottom up influences provided a necessary but not sufficient condition for the illusion to occur. The discussion about the relative influence of bottom-up and top-down factors has continued with the introduction of the ideas of offline and online representations [30] where the former refers to what our body is normally like, and the latter refers to the temporal flow of information that constructs how our body is right now [31], [32]. However, [30] refers to self recognition, which may not be the same as body ownership. We would argue that ownership in the sense meant by the RHI illusion refers only to the feeling that the seen rubber or virtual hand or body appears to be the loci of proprioception and tactile sensation. Knowing that it is an illusion, however, does not extinguish this feeling, which appears to be an automatic response of the brain in dealing with the conflicting sensory information. The results of our experiment lend weight to the view that bottom up sensory integration (or visual capture) can alter the sense of one's body by giving these powerful illusions of temporary transformation in the form (female) and size (somewhat smaller) of the body. On the other hand the virtual body does have a humanoid appearance and we do not know whether the illusion would break down if there were changes in the topology of the body. The work described in [29], where it was shown that a non-humanoid appearance of the arm [29] and left-right reversal does not produce the illusion, suggests that there are clearly limits imposed by top down processing on the type of body and its configuration. The same was found with respect to the body in [7].

Through an IVR a person can see through the eyes and hear through the ears of a virtual body that can be seen to substitute for their own body, and our data show that people have some subjective and physiological responses as if it were their own body. This virtual body may be seen perceived when looking directly at oneself from a first-person perspective (or in a virtual mirror) and the multisensory and sensorimotor contingencies involved in the active process of looking down and seeing a virtual body where one's own body would be provides an important tool not just for presence and virtual reality research [24], but also to understand – eventually in conjunction with neurophysiology and neuroimaging techniques - the neurobiology of self-consciousness.

## Materials and Methods

### Introduction

24 male subjects were recruited who were naïve to the purposes of the experiment. The experiment was approved by the Comité Ético de Investigación del IDIBAPS (Hospital Clínic, University of Barcelona) and written informed consent was obtained from all participants. A balanced between-groups design was used with the three binary factors being Perspective, Movement and Touch as discussed above (Table 1). Subjects were fitted with a wide field-of-view light-weight (less than 1Kg) head-mounted display (HMD) which was head tracked. This was a Fakespace Labs Wide5 HMD, which has field of view 150°×88° with an estimated 1600×1200 resolution displayed at 60Hz, and the head-tracking was with an Intersense 900. They were also fitted with a Nexus 4 physiological recording device. Electrodes attached to this device were placed on their left and right collar bones and the lowest left rib in order to record the ECG (sampling rate 1024Hz).

Once they had entered the virtual environment they were asked to look around the environment and report what they saw, for purposes of acclimatization to the HMD and familiarization with the environment, and especially to see the standing woman and seated girl on the other side of the room. Then the experiment started signaled by the virtual TV screen showing a music video, and there was no further communication with the experimenters until the completion of the trial. At the end of the experiment the participants completed a questionnaire, and the critical questions relating to the issue of body ownership are shown in supporting information Questionnaire S1.

Supporting information can be found in Methods S1 and Movie S1.

### Subject Recruitment

Male participants were recruited by advertisement around the campus. Recruitment continued until we had error free physiological data for the target 24 participants. The mean age of the retained 24 participants was 27.6±4.3 years, almost all were students, researchers or employees of the University, who had no prior knowledge of the experiment. Most of them had no prior experience of virtual reality, and none of them had any experience of our virtual reality system or laboratory. They were paid 10€ for their participation.

### Procedures

After being fitted with the physiological recording equipment and the HMD the subjects were instructed to look around the virtual environment and report what they could see. This was in order for them to become used to wearing the HMD and also to become familiar with the scene, and especially to notice the two female virtual characters on the other side. After this period of acclimatization headphones were put on and the virtual TV screen on the left hand side of the room now started playing a recorded music video. The subjects had been instructed to continue to look around the room, remembering also to look downwards.

After 2 minutes the first viewpoint transition occurred after which the subject was on the other side of the virtual room. How they experienced subsequent events depended on which combination of the experimental factors they had been assigned.

### Experimental Factors

The experimental factors were:

#### Perspective

In the 1PP (first person) condition the participant saw through the eyes of the body of the virtual girl (Figure 2A) and in the 3PP condition (third person) the participant's position was 1m to the left of the girl's body (Figure 2F).

#### Movement

In the synchronous condition (MS) the head movements of the girl were displayed to be synchronous with those of the participant (Figs 2D, 2F); in the asynchronous condition (MS′) the head movements of the girl were displayed to move asynchronously with respect to the participant, and were based on pre-recorded head movements. Although the movements of the virtual girl's head were synchronous or asynchronous with respect to the head movements of the participant, this was independent of the visual field seen by the participant. This was always correct based on his head position and orientation with respect to the virtual scene, and determined wholly by the head-tracking.

#### Touch

In the synchronous condition (TS) when the woman stroked the shoulder of the virtual girl the participant would feel synchronous stroking on his shoulder (visuotactile correlation) (Figure 2C, D,F, G); in the asynchronous condition (TS′) the stroking felt on his shoulder would occur during the same time interval as the stroking seen on the girl but the felt strokes themselves would be asynchronous with respect to the visual strokes.

### Sequence of Events

Various events were programmed to occur during the course of the 10 minutes after the viewpoint transition (Table 2). Almost immediately after ‘arriving’ in the location of the girl (white shirt), the virtual woman (brown sweater) raised her left arm and stroked the right shoulder of the girl. There were 22 stroking periods during the course of the subsequent 10 minute experience, where there was visuotactile stimulation (either synchronous or asynchronous) each consisting of between 1 and 5 strokes. There were 53 such strokes in total and the total duration of all the stroking animations was 217s. There were 5 periods of stroking while the participant experienced the elevated position looking down on the scene, where there was no tactile stimulation associated with the visual stroking. A critical event was that 415s after the first viewpoint transformation there was a second transformation, where the participant's viewpoint was elevated to near the top of the ceiling and oriented to be looking down on the scene below (Figure 2G). The spatial coordinates of this viewpoint transformation were chosen based such transformations in neurological patients with out-of-body experiences [33], [34]. As reported by many of these patients this elevated location is not represented as embodied in a person's body and patients report not perceiving or having reduced perception of somatosensory cues from their body. Accordingly, although the visual arm strokes continued during this period they were not accompanied by any actual stroking of the participant's arm. Then 45s into this elevated position the woman suddenly slapped the girl around the face three times, and the girl's body swayed (Figure 2H) and there were corresponding sounds. We rendered our animation in this way because previous work on ownership has shown that threat-like behavior is often associated with physiological changes [2], [9], [14], [35]. After this, the situation returned to as it had been before the slapping, with the occasional arm stroking, and after a further 50s the perspective shifted down again to the same situation as it had been before the upward translation (location of the girl).

### Questionnaire

The questionnaire administered afterwards is shown in Questionnaire S1. The questionnaire scores (between 0 and 10) were recoded into ranges as Very Low (0), Low (1–3), Medium (4–6), High (7–9) and Very High (10), based on the layout of the questionnaire. Some of the questions were derived from previous work [1], [7], [9] and others were introduced following interviews with participants in extensive pilot trials. We note that questionnaires in this area are problematic unless backed up by behavioral or physiological evidence, see the discussion of this point with respect to the virtual arm illusion in [11].

### Statistical Methods

For the questionnaire responses we have chosen the proportional odds cumulative logit model [36] since this appropriately treats the responses as ordinal data. In order to use this we needed to group scores together, since the responses are too sparsely distributed over the original range of scores from 0 to 10.

However, for comparison we have also analysed the questionnaire responses using traditional analysis of variance. Three-way analyses of variance with all two-way interactions were carried out on the questionnaire responses. This is strictly not an appropriate form of analysis since it assumes that the responses are at least on an interval scale, and clearly this is not the case. Nevertheless this approach is common, and it is presented for the sake of completeness, but the results are not different from the previous analysis. The significant results are presented in Table 5. The residual errors of all models were compatible with normality, based on Jarque-Bera tests.

**Table 5:** Significance Levels of Analysis of Variance of Questionnaire Responses.

Questionnaire Variable	P	T
Hurt	0.041	
Body	0.033	0.100
Touch	0.031	
Cloth	0.0003	

A requirement of ANOVA is that the residual errors of the model fit should follow a normal distribution. In most of the analyses we carried out this was the case, as judged by a Jarque-Bera test [37]. When this is not the case the standard solution is to try to find a monotonic transformation of the response variable so that the residual errors become normal. This was accomplished systematically using the Box-Cox family of transformations [38] 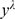, and finding the maximum likelihood estimator for 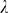.

## Supporting Information

Figure S1 Scatter diagram of the questionnaire responses of mirror by body, classified by M. Some of the coordinates occur more than once so that the plotted points overlay one another. Points above the diagonal line have mirror > body.
(1.97 MB TIF)

Figure S2 Estimated probabilities for the questionnaire responses on the touch related questions. These are shown for four combinations of the factors, each with M = MS. The results are almost identical for MS′. The left panel shows the results for touch (Q5), and the right woman (Q10). There are no 0 scores for touch.
(4.74 MB TIF)

Questionnaire S1 The post experiment questionnaire.
(0.02 MB PDF)

Methods S1 Supporting Methods.
(0.02 MB PDF)

Movie S1 The video shows some extracts from the scenario, mainly from the first person perspective position of the virtual girl, and also from the 3PP condition. There is no sound on the video although the subjects would have heard the music from the virtual TV (spatialised). The sound is not included for copyright reasons.
(20.46 MB MOV)

## References

[1] Botvinick M, Cohen J (1998). Rubber hands ‘feel’ touch that eyes see.. Nature.

[2] Armel KC, Ramachandran VS (2003). Projecting sensations to external objects: evidence from skin conductance response.. Proceedings of the Royal Society of London Series B-Biological Sciences.

[3] Schaefer M, Flor H, Heinze H, Rotte M (2007). Morphing the body: Illusory feeling of an elongated arm affects somatosensory homunculus.. Neuroimage.

[4] Schaefer M, Heinze H, Rotte M (2009). My third arm: Shifts in topography of the somatosensory homunculus predict feeling of an artificial supernumerary arm.. Human Brain Mapping.

[5] Ehrsson H (2009). How many arms make a pair? Perceptual illusion of having an additional limb.. Perception.

[6] Ehrsson H, Kito T, Sadato N, Passingham R, Naito E (2005). Neural Substrate of Body Size: Illusory Feeling of Shrinking of the Waist.. PLoS Biol.

[7] Lenggenhager B, Tadi T, Metzinger T, Blanke O (2007). Video ergo sum: Manipulating bodily self-consciousness.. Science.

[8] Lenggenhager B, Mouthon M, Blanke O (2009). Spatial aspects of bodily self-consciousness.. Consciousness and Cognition.

[9] Ehrsson HH (2007). The experimental induction of out-of-body experiences.. Science.

[10] Ehrsson HH, Spence C, Passingham RE (2004). That's my hand! Activity in premotor cortex reflects feeling of ownership of a limb.. Science.

[11] Slater M, Perez-Marcos D, Ehrsson HH, Sanchez-Vives M (2008). Towards a digital body: The virtual arm illusion.. Front Hum Neurosci.

[12] Perez-Marcos D, Slater M, Sanchez-Vives M (2009). Inducing a virtual hand ownership illusion through a brain-computer interface.. Neuroreport.

[13] Slater M, Perez-Marcos D, Ehrsson HH, Sanchez-Vives MV (2009). Inducing Illusory Ownership of a Virtual Body.. Frontiers in Neuroscience.

[14] Petkova VI, Ehrsson HH (2008). If I Were You: Perceptual Illusion of Body Swapping.. PLoS ONE.

[15] Blanke O, Metzinger T (2009). Full-body illusions and minimal phenomenal selfhood.. Trends in Cognitive Sciences.

[16] Cacioppo J, Tassinary L, Berntson G (2007). Handbook of Psychophysiology.

[17] Vogeley K, Fink G (2003). Neural correlates of the first-person-perspective.. Trends in Cognitive Sciences.

[18] Vogeley K, May M, Ritzl A, Falkai P, Zilles K (2004). Neural correlates of first-person perspective as one constituent of human self-consciousness.. Journal of Cognitive Neuroscience.

[19] Blakemore S, Wolpert D, Frith C (2002). Abnormalities in the awareness of action.. Trends in Cognitive Sciences.

[20] Jeannerod M (2003). The mechanism of self-recognition in humans.. Behavioural Brain Research.

[21] Tsakiris M, Haggard P, Franck N, Mainy N, Sirigu A (2005). A specific role for efferent information in self-recognition.. Cognition.

[22] Lloyd D (2007). Spatial limits on referred touch to an alien limb may reflect boundaries of visuo-tactile peripersonal space surrounding the hand.. Brain and Cognition.

[23] Schutz-Bosbach S, Mancini B, Aglioti SM, Haggard P (2006). Self and other in the human motor system.. Current Biology.

[24] Sanchez-Vives MV, Slater M (2005). From Presence to Consciousness through Virtual Reality.. Nature Reviews Neuroscience.

[25] Noë A (2004). Action In Perception.

[26] O'Regan JK, Noë A (2001). A sensorimotor account of vision and visual consciousness.. Behav Brain Sci.

[27] Slater M (2009). Place Illusion and Plausibility Can Lead to Realistic Behaviour in Immersive Virtual Environments.. Philos Trans R Soc Lond.

[28] Kurzban R, Tooby J, Cosmides L (2001). Can race be erased? Coalitional computation and social categorization.. PNAS.

[29] Tsakiris M, Haggard P (2005). The rubber hand illusion revisited: Visuotactile integration and self-attribution.. Journal of Experimental Psychology-Human Perception and Performance.

[30] Carruthers G (2008). Types of body representation and the sense of embodiment.. Consciousness and Cognition.

[31] Tsakiris M, Fotopoulou A (2008). Is my body the sum of online and offline body-representations?. Consciousness and Cognition.

[32] Carruthers G (2008). Reply to Tsakiris and Fotopoulou ìIs my body the sum of online and offline body representations?íí.. Consciousness and Cognition.

[33] Devinsky O, Feldmann E, Burrowes K, Bromfield E (1989). Autoscopic phenomena with seizures.. Archives of Neurology.

[34] Blanke O, Landis T, Spinelli L, Seeck M (2004). Out-of-body experience and autoscopy of neurological origin.. Brain.

[35] Ehrsson H, Wiech K, Weiskopf N, Dolan R, Passingham R (2007). Threatening a rubber hand that you feel is yours elicits a cortical anxiety response.. Proceedings of the National Academy of Sciences.

[36] McCullagh P, Nelder JA (1989). Generalized linear models.

[37] Jarque CM, Bera AK (1980). Efficient tests for normality, homoscedasticity and serial independence of regression residuals.. Economics Letters.

[38] Box G, Cox D (1964). An analysis of transformations.. Journal of the Royal Statistical Society Series B (Methodological).

