# Supplementary figures and images for: First Person Experience of Body Transfer in Virtual Reality
Source: PLoS One. 2010 May 12;5(5):e10564. doi: 10.1371/journal.pone.0010564 (PMC2868878; doi:10.1371/journal.pone.0010564)

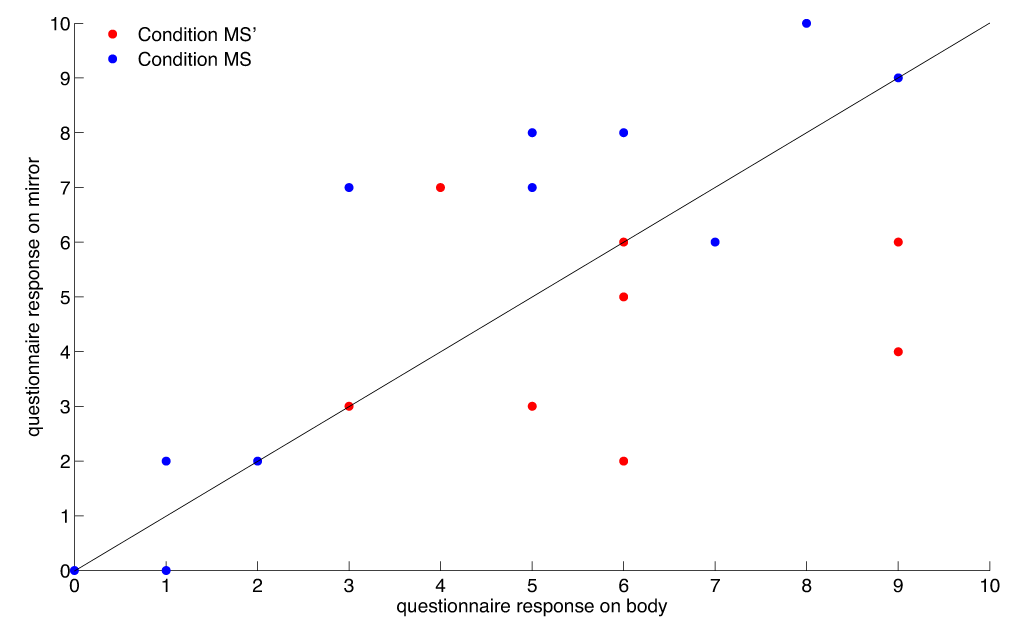

Supplement: Figure S1 — Scatter diagram of the questionnaire responses of mirror by body, classified by M. Some of the coordinates occur more than once so that the plotted points overlay one another. Points above the diagonal line have mirror > body. (1.97 MB TIF) [file pone.0010564.s001.tif]

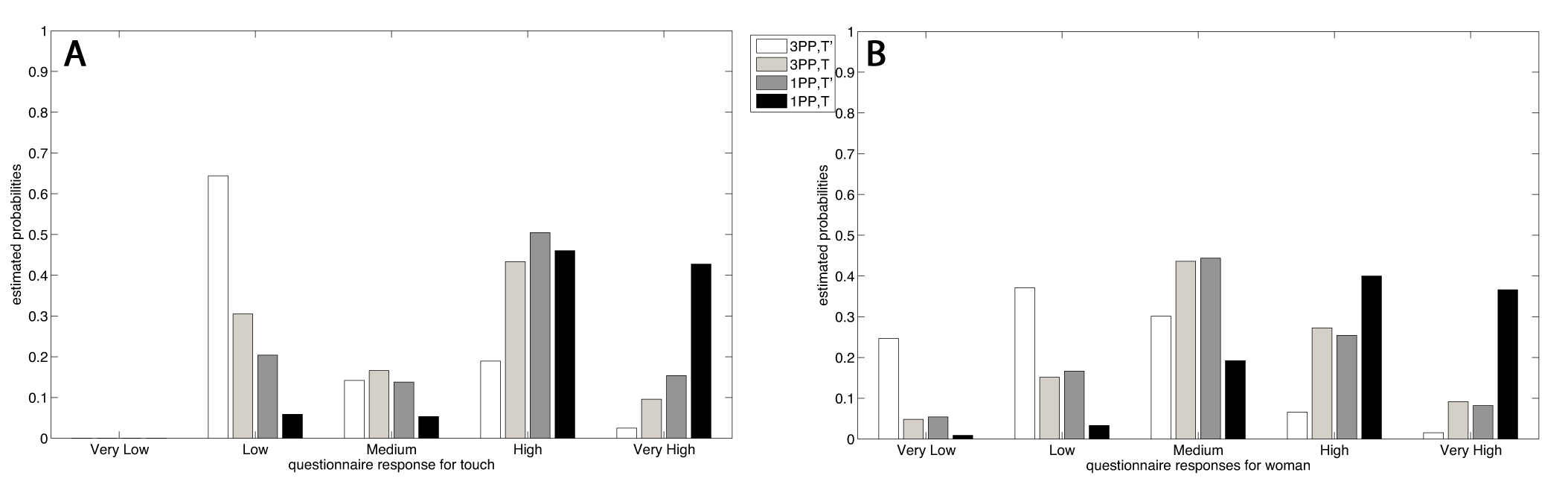

Supplement: Figure S2 — Estimated probabilities for the questionnaire responses on the touch related questions. These are shown for four combinations of the factors, each with M = MS. The results are almost identical for MS′. The left panel shows the results for touch (Q5), and the right woman (Q10). There are no 0 scores for touch. (4.74 MB TIF) [file pone.0010564.s002.tif]
