# Supplementary material for: First Person Experience of Body Transfer in Virtual Reality
Source: PLoS One. 2010 May 12;5(5):e10564. doi: 10.1371/journal.pone.0010564 (PMC2868878; doi:10.1371/journal.pone.0010564)
Supplement: Questionnaire S1 — The post experiment questionnaire. (0.02 MB PDF) [file pone.0010564.s003.pdf]

Questionnaire

1. When you were looking down from above how much did you feel a strong connection with the seated girl as if you were looking down at yourself?

NOT AT ALL	0	1 2 3 4 5 6 7 8 9	10 VERY MUCH

2. When the standing woman hit the seated woman, how much did you feel this as if this was an attack on your body?

NOT AT ALL	0	1 2 3 4 5 6 7 8 9	10 VERY MUCH

3. After you returned from looking down from above how much did you feel that the standing woman might hurt you?

NOT AT ALL	0	1 2 3 4 5 6 7 8 9	10 VERY MUCH

4. How much did you feel that the seated girl's body was your body?

NOT AT ALL	0	1 2 3 4 5 6 7 8 9	10 VERY MUCH

5. How strong was the feeling that the woman you saw was directly touching you on the shoulder?

NOT AT ALL	0	1 2 3 4 5 6 7 8 9	10 VERY MUCH

6. How strong was the feeling that your body had shifted location when you moved to the other side of the virtual room?

NOT AT ALL	0	1 2 3 4 5 6 7 8 9	10 VERY MUCH

7. How much was this more like watching a scene from the outside compared to really being part of the scene?

FROM OUTSIDE	0	1 2 3 4 5 6 7 8 9	10 PART OF THE SCENE

8. How much did you feel heat from the fire that you saw?

NOT AT ALL	0	1 2 3 4 5 6 7 8 9	10 VERY MUCH

9. How strong was the feeling that the body of the girl in the mirror was your body?

NOT AT ALL	0	1 2 3 4 5 6 7 8 9	10 VERY MUCH

10. How strong was the feeling that the touch you felt was caused by the woman that you saw?

NOT AT ALL	0	1 2 3 4 5 6 7 8 9	10 VERY MUCH

11. When you were above the scene how strong was the feeling that you were dissociated from your body (as if your self and your body were in different locations)?

NOT AT ALL	0	1 2 3 4 5 6 7 8 9	10 VERY MUCH

12. After you returned from looking down from above how much did you feel that the standing woman appeared to be different compared to before?

NOT AT ALL	0	1 2 3 4 5 6 7 8 9	10 VERY MUCH

13. How strong was the feeling that you were wearing different clothing, from when you started the experiment, while you were in the part of the room where the standing woman was located?

NOT AT ALL	0	1 2 3 4 5 6 7 8 9	10 VERY MUCH
