# Supplementary material for: First Person Experience of Body Transfer in Virtual Reality
Source: PLoS One. 2010 May 12;5(5):e10564. doi: 10.1371/journal.pone.0010564 (PMC2868878; doi:10.1371/journal.pone.0010564)
Supplement: Methods S1 — Supporting Methods. (0.02 MB PDF) [file pone.0010564.s004.pdf]

SUPPORTING METHODS

First person experience of body transfer in virtual reality

Mel Slater, Bernhard Spanlang, Maria V. Sanchez-Vives, Olaf Blanke

General Procedures

When participants first entered the laboratory they were given an information sheet to read about the experiment, and if they agreed to continue (all did) they signed a consent form. They then completed a short questionnaire that provided basic demographic information (age, status, medicine prescriptions, the extent of game playing, and past experience with virtual reality).

There were two experimenters in the lab throughout. One helped the participant put on the ECG recording equipment. They were then invited to sit in a comfortable position with their hands resting on their knees. This was to roughly match the posture of the virtual girl. They then entered into a virtual reality by donning a Fakespace Labs Wide5 head-mounted display (HMD), which has field of view $150^{\circ} \times 88^{\circ}$ with an estimated 1600×1200 resolution displayed at 60Hz. The lights in the lab were then turned off.

Participants were asked to open their eyes and to look around and to describe all the features of the virtual room. They were told to look around only by moving their head, not by turning their whole upper body. This was because only their head was tracked, and therefore only the head movements of the virtual girl could match their own movements. While looking around they would be able to see to their left a TV, a window, and a bookcase. Directly in front of them was a table with a small Pinocchio figure on it, and in front at the other side of the room was a seated girl facing towards the subject, and facing away from the subject but towards the girl was a standing woman who could be seen to be occasionally stroking the right shoulder of the girl. Nearby could be seen the frame of a body sized mirror facing towards the girl. Behind the girl was a fireplace with a burning fire. To the right was a sofa and on the wall was a painting. As the participants continued looking round to their right they would see a frame for a large mirror, but not the mirror itself, and then an open door to the outside, and some grass could be seen through the door. Participants were instructed to look down at themselves and they would see an empty chair. They were told that once the scenario started they should continue to look around, remembering occasionally to look down at themselves, and that this was their only task.

An experimenter then put some headphones (Sennheiser HD215 stereo) on the participants and initiated the actual start of the experiment. The participants would then see in the virtual reality a video playing on the virtual TV, and would hear associated sounds and music that were directional. This continued for 2 minutes after which the image that they saw distorted giving the impression of movement and when the image reformed their visual ego centre was on the

other side of the room where the virtual females had been seen to be located. What happened next was the same for all participants, except that they would perceive the events differently, depending on the combination of factors to which they had been assigned.

Throughout the whole experiment an experimenter stood behind where the participant was sitting, and paid attention to a PC screen displaying what the participant was seeing. The experimenter was responsible for stroking the arm of the participant whenever the virtual woman stroked the arm of the girl, and these arm strokes could always be seen on the monitor. In the case of the synchronous (T) condition the experimenter would carefully match the touch of the woman, stroke for stroke, and in asynchronous (T') condition would deliver strokes during the same period as the woman, but being careful not to match the woman's strokes.

Finally the participant's viewpoint was moved back to the original starting position on the original side of the room, and the experiment was complete. The lab lights were switched on.

Participants were asked for their immediate impressions of their experience while they were taking off the various equipment, and then asked to complete the questionnaire (available in Spanish and English) (Table S3).

Physiological Measures

The ECG was acquired with the Nexus 4 device (sampling frequency: 1024 Hz)**,** and the analysis was performed with the g.BSanalyze biosignal analysis software package (g.tec – Guger Technologies OEG, Graz, Austria). The first step in ECG analysis was to detect QRS (ventricular contraction) complexes in the ECG time series. The QRS complexes determine the distance in time from one heart contraction to the next one (RR interval). The QRS complexes in the ECG data were detected automatically based on a modified Pan-Tompkins algorithm [1]. Then a visual inspection of the detected QRS complexes was performed to correct any missed or wrongly assigned points.

1. Pan J, Tompkins WJ (1985) A Real-Time Qrs Detection Algorithm. Ieee Transactions on Biomedical Engineering 32: 230-236.
